# Safety of High-Dose Vitamin D Supplementation Among Children Aged 0 to 6 Years: A Systematic Review and Meta-analysis

**DOI:** 10.1001/jamanetworkopen.2022.7410

**Published:** 2022-04-14

**Authors:** Nicklas Brustad, Sina Yousef, Jakob Stokholm, Klaus Bønnelykke, Hans Bisgaard, Bo Lund Chawes

**Affiliations:** 1 COPSAC (Copenhagen Prospective Studies on Asthma in Childhood), Herlev and Gentofte Hospital, University of Copenhagen, Gentofte, Denmark; 2 Section of Microbiology and Fermentation, Department of Food Science, University of Copenhagen, Copenhagen, Denmark

## Abstract

**Importance:**

Several health benefits of vitamin D have been suggested; however, the safety of high-dose supplementation in early childhood is not well described.

**Objective:**

To systematically assess the risk of adverse events after high-dose supplementation with vitamin D reported in published randomized clinical trials.

**Data Sources:**

PubMed and ClinicalTrials.gov were searched through August 24, 2021.

**Study Selection:**

Randomized clinical trials of high-dose vitamin D supplementation in children aged 0 to 6 years, defined as greater than 1000 IU/d for infants (aged 0-1 year) and greater than 2000 IU/d for children aged 1 to 6 years.

**Data Extraction and Synthesis:**

Following the Preferred Reporting Items for Systematic Reviews and Meta-analyses (PRISMA) reporting guideline, 2 reviewers independently extracted the data from the eligible studies. Summary risk ratio (RR), 95% CI, and *P* values were derived from random-effects meta-analysis.

**Main Outcomes and Measures:**

Adverse events, serious adverse events (SAEs), and/or levels of 25-hydroxyvitamin D, calcium, alkaline phosphatase, phosphate, parathyroid hormone, and/or the ratio of urine calcium to creatinine levels.

**Results:**

A total of 32 randomized clinical trials with 8400 unique participants were included. Different clinical outcomes of children receiving high-dose vitamin D supplements ranging from 1200 to 10 000 IU/d and bolus doses from 30 000 IU/week to a single dose of 600 000 IU were evaluated. Eight studies with 4612 participants were eligible for meta-analysis using a control group receiving either low-dose vitamin D supplementation (≤400 IU/d) or placebo when investigating the risk of SAEs such as hospitalization or death. No overall increased risk of SAEs in the high-dose vitamin D vs control groups was found (RR, 1.01 [95% CI, 0.73-1.39]; *P* = .89, *I*^2^ = 0%). In addition, risk of hypercalcemia (n = 726) was not increased (RR, 1.18 [95% CI, 0.72-1.93]; *P* = .51). Clinical adverse events potentially related to the vitamin D supplementation reported in the studies were rare.

**Conclusions and Relevance:**

This meta-analysis and systematic review found that high-dose vitamin D supplementation was not associated with an increased risk of SAEs in children aged 0 to 6 years, and that clinical adverse events potentially related to the supplementation were rare. These findings suggest that vitamin D supplementation in the dose ranges of 1200 to 10 000 IU/d and bolus doses to 600 000 IU to young children may be well tolerated.

## Key Points

**Question:**

Is high-dose vitamin D supplementation safe for children aged 0 to 6 years?

**Findings:**

In this systematic review and meta-analysis of 32 randomized clinical trials including 8400 unique children, high-dose vitamin D administered as a daily or bolus supplement was not associated with an increased risk of serious adverse events. Clinical adverse events associated with the supplementation were rare.

**Meaning:**

This systematic review and meta-analysis suggests that vitamin D supplements in daily doses to 10 000 IU/d or bolus doses to 600 000 IU are well tolerated in children aged 0 to 6 years.

## Teaser

This systematic review and meta-analysis investigates the safety of high-dose vitamin D supplementation in children aged 0 to 6 years by examining potential clinical adverse events and biochemical changes reported in randomized clinical trials.

## Introduction

The influence of vitamin D on skeletal health is well known, but other biological nonskeletal effects have been discovered as the vitamin D receptor has been identified in cell types not involved in bone metabolism.^1,2,3^ Vitamin D deficiency is a common global problem,^3^ particularly in young children,^4^ and the insight about new biological nonskeletal actions of vitamin D demands revision of vitamin D supplementation policies,^1^ which have led to updated recommendations regarding clinical practice and recommended intake levels. Vitamin D is primarily obtained by cutaneous synthesis from sunlight exposure or as a nutrient from dietary sources such as fatty fish.^5^ However, as treatment and in prevention of deficiency, vitamin D supplements in the form of either cholecalciferol (vitamin D_3_) or ergocalciferol (vitamin D_2_) have been recommended.^6^ Supplementation of very high doses of vitamin D can cause hypercalcemia and subsequent kidney failure; therefore, an upper threshold concentration of serum 25-hydroxyvitamin D (25[OH]D) corresponding to 100 ng/mL (to convert to nanomoles per liter, multiply by 2.496) has been suggested by the Endocrine Society, at which the risk of developing hypercalcemia is considered minimal.^6^ Regarding intake, a tolerable upper intake level has been defined as 25 μg/d (1000 IU/d) for infants aged 0 to 12 months and 50 μg/d (2000 IU/d) for children aged 1 to 10 years by the European Food Safety Authority.^3^ In general, evidence from systematic reviews and meta-analyses evaluating the safety of high-dose vitamin D supplementation in early childhood is lacking. Therefore, the purpose of this study was to investigate the safety of high-dose vitamin D in children aged 0 to 6 years by examining potential clinical adverse events and biochemical changes reported in randomized clinical trials (RCTs).

## Methods

This study was conducted in accordance with the Preferred Reporting Items for Systematic Reviews and Meta-analyses (PRISMA) guidelines. This study was registered with PROSPERO (CRD42020204074).

### Search Strategy

A systematic literature search was conducted using the PubMed database to identify available studies until August 24, 2021. The search was restricted to English-language studies. Search terms were divided into 3 aspects using the following key terms: *randomized controlled trials*, *child/infant*, and *vitamin D* (eFigure 1 in the Supplement). The search protocol is provided in detail in eTable 1 in the Supplement. Both MeSH (Medical Subject Headings) and text word terms were used in the combined searches. Boolean operators were used to specify the search. All hits were screened based on title and abstract, and inclusion of the final studies was based on full-text reading and consensus with the coreviewers.

### Study Selection and Quality Assessment

The study selection was based on the following inclusion criteria: (1) original RCT, (2) intervention with vitamin D_2_ or D_3_ supplementation greater than 1000 IU/d or bolus therapy, (3) children aged 0 to 6 years, and (4) clinical adverse events and/or biochemical levels of 25(OH)D, calcium, alkaline phosphatase (ALP), phosphate, parathyroid hormone (PTH), and/or ratio of urine calcium to creatinine levels (Ca:Cr ratio) must have been shown in figures or described in tables or the text. Exclusion criteria consisted of (1) secondary follow-up articles (ie, not primary RCT article), (2) topical administration of vitamin D, and (3) coadministration of calcium supplementation. All studies were quality assessed using the Cochrane Risk of Bias Tool.^7^

### Data Extraction and Synthesis

Two authors (N.B. and S.Y.) extracted the following information from the selected studies: first author, publication year, outcomes, total sample size, description of interventions, race and ethnicity, and details of the study participants. Information on safety outcomes extracted included the number of children with 25(OH)D levels greater than 100 ng/mL; hypercalcemia; abnormal levels of ALP, phosphate, PTH, and/or Ca:Cr ratio; and clinical and serious adverse events (SAEs). The data were extracted directly from the selected articles and supported by information from the registration site (eg, ClinicalTrials.gov). If several follow-up periods with biochemical measures were presented in the articles, the follow-up period with the highest biochemical values regarding 25(OH)D, calcium, phosphate, and Ca:Cr ratio and the lowest ALP and PTH levels were extracted and used when assessing changes from baseline measurements.

The reference ranges and comments from the authors of the included studies were used to categorize biochemical values as abnormal or within reference ranges. In this study, high-dose vitamin D supplementation was defined as greater than 1000 IU/d for children aged 0 to 1 year and greater than 2000 IU/d for children aged 1 to 6 years based on the European Food Safety Authority’s defined upper levels of tolerable intake.^3^

### Statistical Analysis

We performed a meta-analysis of the extracted data from eligible articles with an intervention and a control group and calculated the summary risk ratio (RR), 95% CI, and *P* value from a random-effects meta-analysis supported by forest and funnel plots with a linear regression test of asymmetry. The presence of heterogeneity was measured with Cochran *Q* test. The variation in effect size due to between-study heterogeneity was considered using the *I^2^* value. We performed an overall analysis of the risk of SAEs and subgroup analyses by intervention methods. All analyses were performed using the meta package in R, version 4.0.3 (R Program for Statistical Computing). The results were considered statistically significant with 2-sided *P* < .05.

## Results

**Figure 1.**
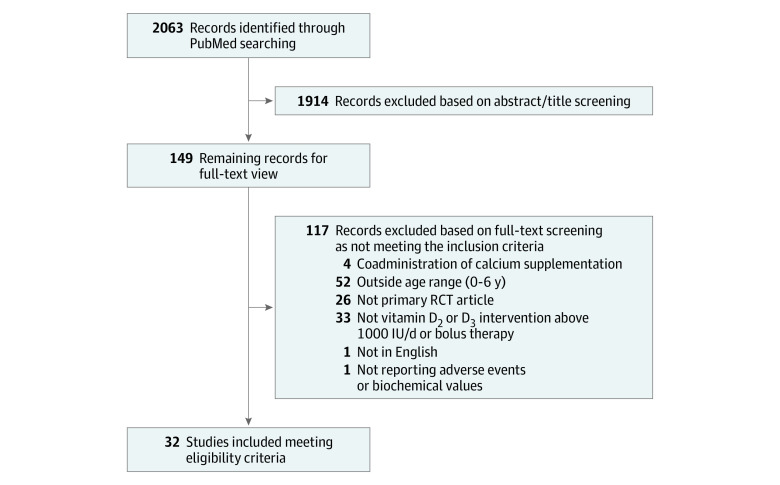
Study Flowchart RCT indicates randomized clinical trial.

The main search resulted in 2063 studies potentially relevant for inclusion (Figure 1); of these, 32 studies^8,9,10,11,12,13,14,15,16,17,18,19,20,21,22,23,24,25,26,27,28,29,30,31,32,33,34,35,36,37,38,39^ (8400 unique participants) met our criteria and were included in this systematic review (Table 1). A subgroup of 21 studies^8,9,10,18,19,20,21,22,23,24,25,28,29,30,31,32,33,34,35,36,37^ (7358 unique participants) were eligible for meta-analysis because they included a control group receiving either low-dose vitamin D supplementation (≤400 IU/d) or placebo.

**Table 1.** Study Characteristics

Source (country)	Study period	Outcomes	No. of participants	Intervention and follow-up	Race or ethnicity	Participant age	Diagnosis
Singh et al,^8^ 2019 (India)	January 2013 to September 2014	Recurrent pneumonia	100	Vitamin D bolus of 300 000 IU every 3 mo vs placebo for 1 y	Asian	0-5 y	Pneumonia
Saleem et al,^9^ 2018 (Pakistan)	June 2015 to November 2016	Anthropometric measurements including weight gain and 25(OH)D, calcium, albumin, and prealbumin levels	194	Vitamin D_3_ bolus of 200 000 IU 2 and 4 wk vs placebo; follow-up at 8 wk	Asian	6-59 mo	Severe acute malnutrition
Ducharme et al,^10^ 2019 (Canada)	September 2014 to July 2016	25(OH)D levels, hypercalciuria, Ca:Cr ratio, asthma treatment, and hospitalizations	47	Vitamin D_3_ bolus of 100 000 IU vitamin D_3_ at baseline and after 3.5 mo vs placebo; follow-up until 7 mo	Multiracial with mostly White	1-5 y	Asthma
Moslemi et al,^11^ 2018 (Iran)	April to August 2016	25(OH)D levels and adverse effects including measurement of biochemical values	108	Vitamin D_3_, 50 000 IU twice per week for 3 wk vs single IM dose of 300 000 IU; follow-up at 3 wk	Asian	30-72 mo	Vitamin D deficiency
Harnot et al,^12^ 2017 (India)	July 2012 to June 2013	Hypercalciuria, hypercalcemia, and 25(OH)D sufficiency	60	Single oral dose of vitamin D, 600 000 vs 300 000 IU; follow-up at 7-10 d	Asian	3 mo to 3 y	Vitamin D deficiency
Mittal et al,^13^ 2014 (India)	November 2010 to April 2012	25(OH)D levels, radiological findings, PTH and ALP levels, hypercalcemia, and Ca:Cr ratio	76	Single oral dose of Vitamin D_3_, 300 000 to 600 000 IU; follow-up at 12 wk	Asian	6 mo to 5 y	Rickets
Mondal et al,^14^ 2014 (India)	November 2009 to March 2011	Hypercalcemia, hypercalciuria, and 25(OH)D, ALP, calcium, and phosphate levels	71	Single IM dose of vitamin D, 600 000 IU vs oral dose of 60 000 IU weekly for 10 wk; follow-up after 12 wk	Asian	0.5 mo to 5 y	Rickets
Lubani et al,^15^ 1989 (Kuwait)	June 1981 to August 1986	Vitamin D deficiency	250	Dose of vitamin D, 600 000 IU plus 400 IU/d for 6 mo to 1 y vs 2000 IU/d for 4 wk plus 400 IU/d until age 2 y or 1 y after treatment start for older children; follow-up until 24-30 mo of age	Mixed	1 mo to 2 y	Rickets
Zeghoud et al,^16^ 1994 (Algeria)	1991 to 1992	25(OH)D, ALP, calcium, and phosphorus levels	30	Single oral dose of vitamin D, 200 000 IU at birth vs 100 000 IU at birth and 3 and 6 mo; follow-up until 6-9 mo of age	North African	Neonates	Healthy
Mittal et al,^17^ 2018 (India)	NR	Rickets, hypercalcemia, Ca:Cr ratio, and 25(OH)D, PTH, and ALP levels	110	Single oral dose of vitamin D, 90 000 vs 300 000 IU; follow-up at 1 wk and 4 and 12 mo	Asian	6 mo to 5 y	Rickets
Gupta et al,^18^ 2016 (India)	August 2012 to January 2015	Pneumonia and 25(OH)D and PTH levels	324	Single oral dose of vitamin D, 100 000 IU vs placebo; follow-up of blood level measurement at 2 wk	Asian	6 mo to 5 y	Pneumonia
Somnath et al,^19^ 2017 (India)	March 2013 to April 2014	Length of hospital stay in children with acute lower respiratory tract infection and 25(OH)D levels	156	Single oral dose of vitamin D, 100 000 IU vs placebo; follow-up at 72 h for 25(OH)D level	Asian	2 mo to 5 y	Acute lower respiratory infection
Huynh et al,^20^ 2017 (Australia)	August 2013 to May 2014	Vitamin D sufficiency, hypercalcemia, craniotabes, and bone development	70	Vitamin D_3_ of 400 IU/d for 4 mo vs single oral dose of 50 000 IU	Mixed	Newborn infants	Newborn infants of mothers with vitamin D deficiency
Moodley et al,^21^ 2015 (Mexico)	July 2011 to July 2012	Changes in 25(OH)D levels	51	Single oral dose of vitamin D, 50 000 IU vs placebo; follow-up until 6 mo	Hispanic	Infants	Healthy
Jensen et al,^22^ 2016 (Canada)	November 2013 to August 2014	Changes in 25(OH)D levels and vitamin D sufficiency	22	Oral single dose of vitamin D, 100 000 IU vs placebo; follow-up until 6 mo	Mixed with mostly White	1-5 y	Asthma
Manaseki-Holland et al,^23^ 2012 (Afghanistan)	November to May 2009	Incidence and severity of pneumonia	3046	Vitamin D, 100 000 IU every 3 mo for 18 mo vs placebo	Asian	1-11 mo	High-risk pneumonia
Manaseki-Holland et al,^24^ 2010 (Afghanistan)	December 2006 to May 2007	Pneumonia length and risk of repeated episodes	453	Single oral dose of vitamin D, 100 000 IU vs placebo; follow-up until 90 d	Asian	1-36 mo	Pneumonia
Shakiba et al,^25^ 2010 (Iran)	January to September 2007	Changes in 25(OH)D and calcium levels	120	Vitamin D, 200 vs 400 IU/d vs 50 000 IU every 2 mo for 6 mo	Asian	Infants	Healthy
Mawer et al,^26^ 1986 (England)	NR	25(OH)D levels	38	Vitamin D_2_, 1000 IU/d vs 3000 IU/d for 6 wk	European and Asian	Infants (gestational age 25-32 wk)	Premature
Moya et al,^27^ 1977	NR	25(OH)D, calcium, phosphate, ALP, and urine pH levels	35	25(OH)D, 6000 IU/d, vs vitamin D_3_, 6000 IU/d, vs 25(OH)D, 3000 IU/d; all participants received interventions in 20 d	European	3-18 mo	Rickets
Rosendahl et al,^28^ 2018 (Finland)	January 2013 to November 2017	Bone strength and risk of infections	975	Vitamin D_3_, 1200 vs 400 IU/d, from 2 wk to 2 y of age	Scandinavian	Infants	Healthy
Choudhary et al,^29^ 2012 (India)	NR	Length of severe pneumonia	200	Vitamin D, 1000 IU/d at <1 y and 2000 IU/d at >1 y vs placebo for 5 d	Asian	2 mo to 5 y	Severe pneumonia
Gallo et al,^30^ 2013 (Canada)	March 2007 to December 2011	Vitamin D sufficiency (>300 ng/mL)	132	Vitamin D, 400 vs 800 vs 1200 vs 1600 IU/d; all participants received interventions for 11 mo with follow-up until 12 mo	Mixed	1 mo	Healthy
Holmlund-Suila et al,^31^ 2012 (Finland)	September 2010 to February 2011	Vitamin D sufficiency (>320 ng/mL), calcium homeostasis, and skeletal parameters via peripheral quantitative computed tomography	113	Vitamin D, 400 vs 1200 vs 1600 IU/d from 2 wk to 3 mo of age	Scandinavian	2 wk	Healthy
Evans et al,^32^ 1989 (Canada)	NR	Risk of bone disease	81	Vitamin D_2_, 2000 vs 400 IU/d for 6 wk	NR	Infants	Very low birth weight
Aglipay et al,^33^ 2017 (Canada)	September 2011 to June 2015	Viral upper respiratory tract infections	703	Vitamin D, 2000 vs 400 IU/d for a minimum of 4 mo	Mixed	1-5 y	Healthy
Zhou et al,^34^ 2018 (China)	September 2016 to July 2018	Prevention of influenza A and levels of 25(OH)D, calcium, and phosphorus	400	Vitamin D, 1200 vs 400 IU/d for 4 mo	Asian	3-12 mo	Healthy
Tau et al,^35^ 1986 (France)	October 1983 to October 1984	Hypercalcemia and 25(OH)D and thyroid hormone levels in infants with hypothyroidism	25	Vitamin D_2_, 1200 IU/d vs placebo for 6 mo	NR	Infants	Infants with congenital hypothyroidism
Pacheco-Acosta et al,^36^ 2020 (Chile)	August 2015 to July 2016	25(OH)D levels	65	Single oral dose of vitamin D, 100 000 IU vs 400 IU/d; follow-up at 6 mo of age	NR	Infants	Healthy
Aldaghi et al,^37^ 2020 (Iran)	August to November 2018	Atopic dermatitis	81	Vitamin D, 1400 vs 400 IU/d for 2 mo	NR	Infants	Atopic dermatitis
Chowdhury et al,^38^ 2021 (Bangladesh)	June 2014 to June 2018	Length of severe pneumonia	197	Oral single dose of vitamin D, 20 000 IU (<6 mo), 50 000 IU (6-12 mo), or 100 000 IU (13-59 mo) plus 10 000 IU/d after vs placebo plus 10 000 IU/d after for the next 4 d	NR	2-59 mo	Severe pneumonia
Saluja et al,^39^ 2021 (India)	November 2018 to April 2020	25(OH)D, calcium, phosphate, ALP, and PTH levels	66	Vitamin D, 2000 IU/d (3-12 mo) or 4000 IU/d (1-5 y) for 12 wk vs oral single dose of 60 000 IU (3-12 mo) or 150 000 (1-5 y)	NR	3-5 mo	Rickets

Abbreviations: ALP, alkaline phosphatase; Ca:Cr, calcium to creatinine; IM, intramuscular; NR, not reported; PTH, parathyroid hormone; 25(OH)D, 25-hydroxyvitamin D.

SI conversion factor: To convert 25(OH)D to nanomoles per liter, multiply by 2.496.

### Study Characteristics

Daily oral high-dose vitamin D intervention ranged from 1200 to 10 000 IU/d, whereas bolus doses ranged from 30 000 IU/week to 600 000 IU as a single dose. Twenty-five of the 32 RCTs^8,9,10,11,12,13,14,15,16,17,18,19,20,21,22,23,24,26,28,32,33,34,35,36,37^ had 2 intervention groups, whereas the remaining 7 studies^25,27,29,30,31,38,39^ compared more than 2 groups. Only 1 study^11^ used 6 years as the upper age limit in their eligibility criteria. Fifteen studies^16,20,21,23,25,26,28,30,31,32,34,35,36,37,39^ included infants (aged 0-12 months) as their only eligible age group, when they were included. In 9 studies,^8,9,11,12,13,14,15,16,17^ at least 1 intervention group received a bolus dose of greater than 100 000 IU. Intervention doses of less than 2000 IU/d as highest vitamin D supplementation given were only seen among infants, which corresponds to a high-dose intervention in this age group. Most studies had a clinical diagnosis or vitamin D deficiency as inclusion criteria, including rickets in 6 studies,^13,14,15,17,27,39^ pneumonia in 6 studies,^8,18,23,24,29,38^ vitamin D deficiency in 2 studies,^11,12^ asthma in 2 studies,^10,22^ and congenital hypothyroidism,^35^ acute lower respiratory infection,^19^ malnutrition,^9^ atopic dermatitis,^37^ vitamin D–deficient mothers,^20^ prematurity,^26^ and very low birth weight^32^ each in 1 study. The remaining 9 studies^16,21,25,28,30,31,33,34,36^ included healthy participants. The most common race and ethnicity represented in the RCTs was Asian, in 14 studies.^8,9,11,12,13,14,17,18,19,23,24,25,29,34^

### 25(OH)D Level

Serum 25(OH)D levels above the threshold of 100 ng/mL were observed in 11 studies^10,11,14,17,19,20,22,23,25,30,39^ (Table 2). In 5 studies^19,20,23,25,30^ comparing high-dose vitamin D supplementation with a control condition (low dose of ≤400 IU/d or placebo), the total number of cases with levels greater than the 100-ng/mL threshold was 22 of 1682 (1.3%) in the high-dose groups vs 0 of 1702 in the control groups (*P* < .001). Any clinical adverse events possibly associated with the vitamin D intervention as described by the authors were reported in 3 studies^20,25,29^ and included mild symptoms such as diarrhea, vomiting, and irritability. Other clinical adverse events reported in the studies were considered associated with the diagnoses of the participants by the authors (ie, not associated with the vitamin D supplementation).

**Table 2.** Safety Outcomes

Source	Elevated 25(OH)D levels (>100 ng/mL)^a^	Other related biochemical changes	SAEs (death or hospitalization)	High-dose intervention vs control (≤400 IU/d or placebo)
Singh et al,^8^ 2019	NR	NR	Vitamin D vs placebo: 11 of 46 vs 15 of 45 hospitalized	Yes
Saleem et al,^9^ 2018	0	No difference in calcium, albumin, or prealbumin levels	1 participant died due to gastroenteritis (before receiving any intervention)	Yes
Ducharme et al,^10^ 2019	Intervention group, 6 of 23 children with >90 ng/mL, 1 associated with Ca:Cr ratio of >1.0; placebo group, 0 of 24	Abnormal urinary Ca:Cr ratio in 9 of 104 samples (intervention) vs 12 of 117 samples (placebo)	Vitamin D vs placebo: 0 of 23 vs 1 of 24 hospitalized	Yes
Moslemi et al,^11^ 2018	Total of 6 children, 4 in single-dose group and 2 in capsule group	NR	0	No
Harnot et al,^12^ 2017	0	Hypercalcemia and abnormal urinary Ca:Cr ratio (after 7-10 d) in 5 of 27 vs 3 of 28 and abnormal urinary Ca:Cr ratio in 5 of 27 vs 2 of 28 (after 3-5 d)	0	No
Mittal et al,^13^ 2014	0	Hypercalcemia: 1 (300 000 IU) vs 1 (600 000 IU), normal urinary Ca:Cr ratio	0	No
Mondal et al,^14^ 2014	Oral group, 2 of 30; IM group, 1 of 31	Normal urinary Ca:Cr ratio and calcium level after intervention	NR	No
Lubani et al,^15^ 1989	NR	Normal calcium, phosphate, and ALP levels	0	No
Zeghoud et al,^16^ 1994	0	Normal calcium levels	NR	No
Mittal et al,^17^ 2018	Group A (90 000 IU), 0 of 55; group B (300 000 IU), 2 of 55 with >150 ng/mL	Abnormal Ca:Cr ratio: 3 of 55 in group A vs 5 of 55 in group B; hypercalcemia: 3 of 55 in group A vs 2 of 55 in group B	0	No
Gupta et al,^18^ 2016	NR	Normal calcium levels	Vitamin D vs placebo: 19 of 156 vs 20 of 159	Yes
Somnath et al,^19^ 2017	Intervention group, 1 of 78; placebo group, 0 of 76	NR	0	Yes
Huynh et al,^20^ 2017	High-dose group, 2 of 34; 400 IU/d group, 0 of 36	Bolus vs daily low dose: 2 of 34 vs 7 of 36 with hypercalcemia	3 children in all (groups not specified)	Yes
Moodley et al,^21^ 2015	NR	NR	NR	Yes
Jensen et al,^22^ 2016	Intervention group, 2 of 11 with >90 ng/mL; placebo group, 0 of 11	Abnormal Ca:Cr ratio: 1 of 11 vs 1 of 11	0	Yes
Manaseki-Holland et al,^23^ 2012	Vitamin D group, 2 of 1524; placebo group, 0 of 1522	NR	Vitamin D vs placebo: 10 of 1524 vs 7 of 1522	Yes
Manaseki-Holland et al,^24^ 2010	NR	NR	Vitamin D vs placebo: 2 of 224 vs 1 of 229 died	Yes
Shakiba et al,^25^ 2010	Bolus group (50 000 IU every 2 mo), 2 of 30; low-dose groups, 0 of 35	Normal calcium levels	0	Yes
Mawer et al,^26^ 1986	NR	NR	NR	No
Moya et al,^27^ 1977	NR	NR	NR	No
Rosendahl et al,^28^ 2018	0	Hypercalcemia: 32 of 364 in 1200-IU vs 27 of 362 in 400-IU groups; mean PTH level: 16.5 vs 19 pg/mL (*P* = .004)	NR	Yes
Choudhary et al,^29^ 2012	NR	NR	Vitamin D vs placebo: 1 of 87 vs 1 of 86 died	Yes
Gallo et al,^30^ 2013	Group receiving 1600 IU/d, 15 of 16; group receiving 400 IU/d, 0 of 33	Suspected hypercalcemia: 2 in the 800-IU/d, 2 in the 1200-IU/d, and 2 in the 1600-IU/d groups; suspected abnormal Ca:Cr ratio: 1 in the 800-IU/d, 1 in the 1200-IU/d, and 1 in the 1600-IU/d groups	0	Yes
Holmlund-Suila et al,^31^ 2012	Group receiving 1600 IU/d, 1 of 37; group receiving 1200 IU/d, 1 of 38; group receiving 400 IU/d, 0 of 38 with >90 ng/mL	No differences in calcium or PTH levels and urine Ca:Cr ratio	0	Yes
Evans et al,^32^ 1989	NR	No difference in calcium, ALP, or phosphate levels; increased urinary Ca:Cr ratio in control group (*P* < .001)	4 of 45 in 2000-IU group vs 2 of 42 in 400-IU group died or had severe jaundice	Yes
Aglipay et al,^33^ 2017	NR	No differences in calcium, ALP, or PTH levels	0	Yes
Zhou et al,^34^ 2018	NR	NR	7 of 200 in 1200-IU/d group vs 8 of 200 in 400-IU/d group secondary bacterial infection and hospitalized	Yes
Tau et al,^35^ 1986	NR	Increased calcium levels in intervention group; no differences in phosphorus or ALP levels	NR	Yes
Pacheco-Acosta et al,^36^ 2020	0	NR	NR	Yes
Aldaghi et al,^37^ 2020	NR	NR	0	Yes
Chowdhury et al,^38^ 2021	0	No differences in calcium and ALP levels	1 of 97 in high-dose group vs 5 of 100 in placebo group died during hospitalization	No
Saluja et al,^39^ 2021	Daily group, 3 of 33; depot group, 1 of 33	No hypercalcemia; hypophosphataemia: 1 of 33 vs 0 of 33 cases; ALP levels increased in 2 of 33 vs 2 of 33 cases; hyperparathyroidism in 3 of 33 vs 1 of 33 cases (no statistically significant differences)	NR	No

Abbreviations: ALP, alkaline phosphatase; Ca:Cr, calcium to creatinine; IM, intramuscular; NR, not reported; PTH, parathyroid hormone; 25(OH)D, 25-hydroxyvitamin D; SAE, serious adverse event.

SI conversion factors: To convert 25(OH)D to nanomoles per liter, multiply by 2.496; to convert PTH to nanograms per liter, multiply by 1.

^a^ Zero indicates no cases were reported in the study.

### Serious Adverse Events

**Figure 2.**
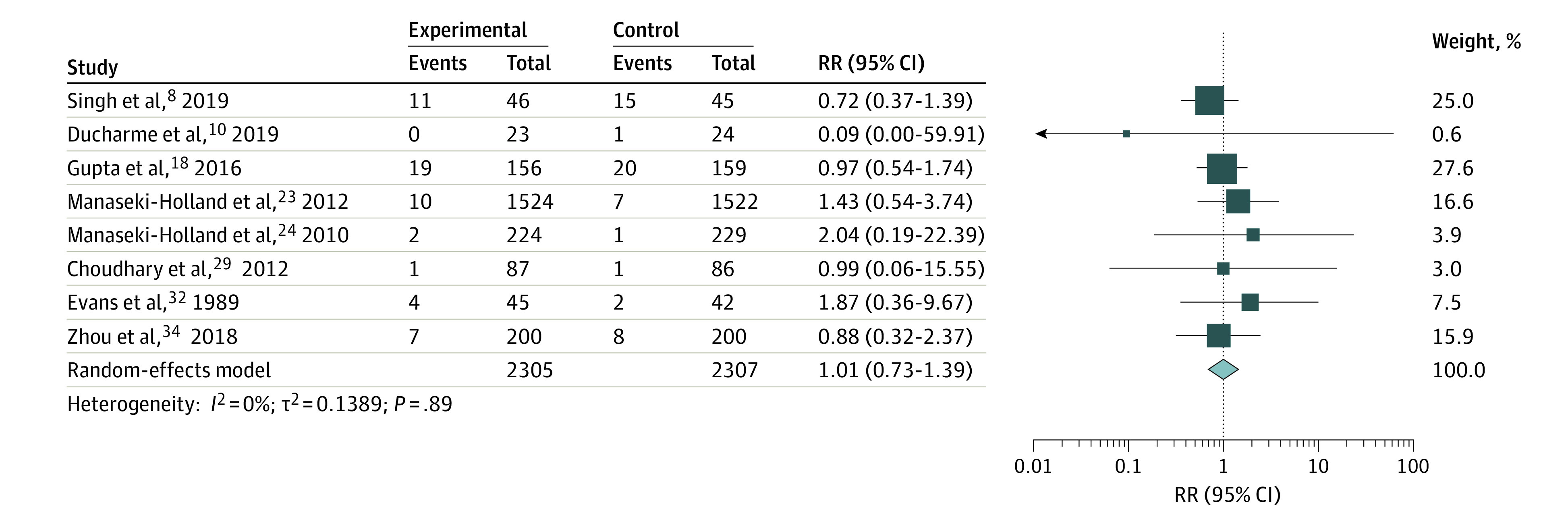
Summary Risk Ratio (RR) of the Association Between High-Dose Vitamin D Supplementation and Serious Adverse Events Different sizes of markers indicate weights of the studies; whiskers, 95% CIs.

**Figure 3.**
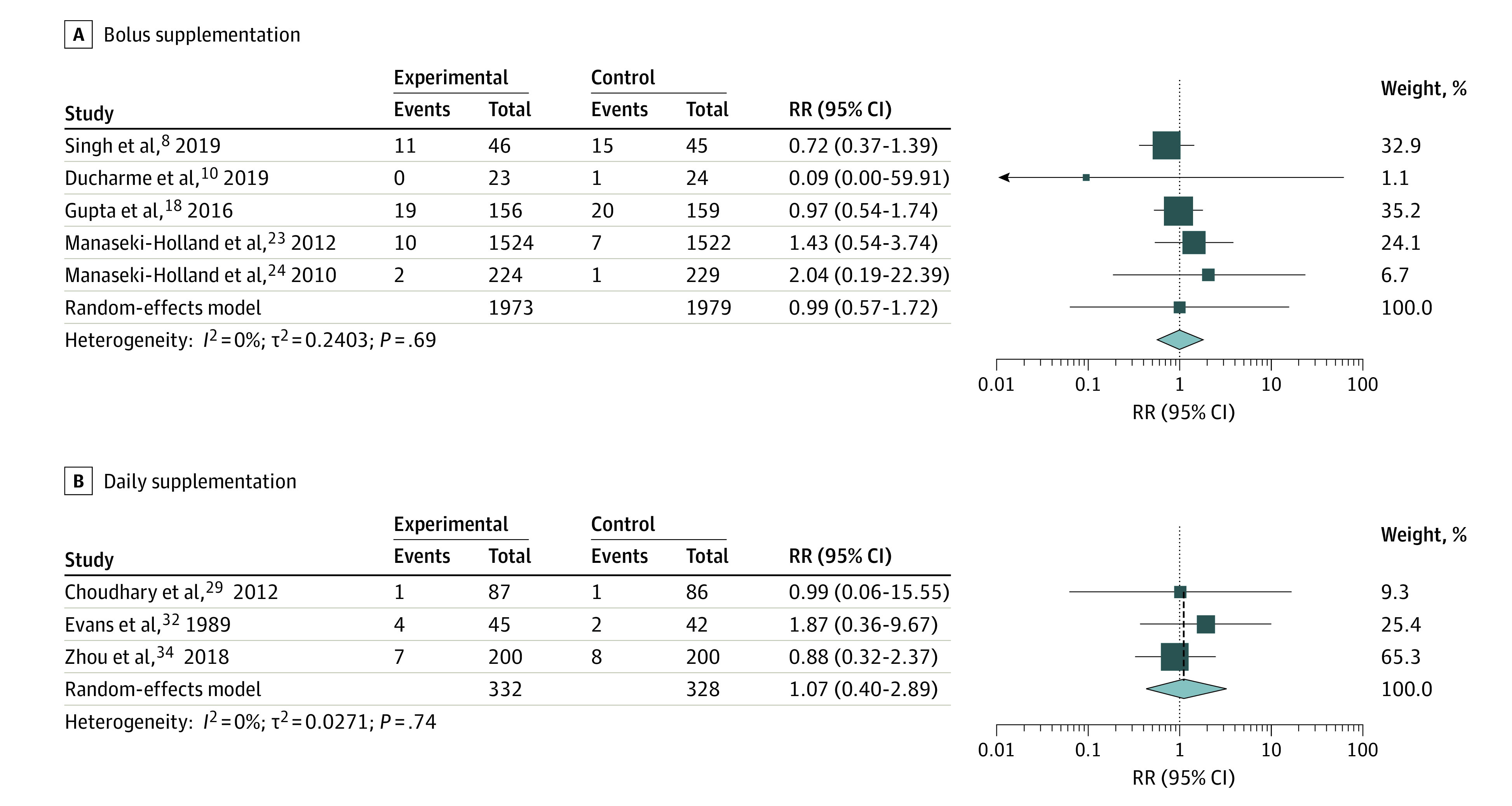
Summary Risk Ratio (RR) of the Association Between High-Dose Vitamin D Supplementation and Serious Adverse Events Stratified by Bolus and Daily Supplementation Different sizes of markers indicate weights of the studies; whiskers, 95% CIs.

Serious adverse events (death or hospitalization) were observed in 10 studies,^8,9,10,18,20,23,24,29,32,34^ of which 8 studies^8,10,18,23,24,29,32,34^ were available for a meta-analysis because 1 study^9^ reported death before receiving any intervention and 1 study^20^ did not specify the group assignments of the 3 children experiencing SAEs. We unsuccessfully tried contacting the authors of the latter study for clarification. There was no increased risk of SAEs in the high-dose vitamin D vs control groups (Figure 2) (RR, 1.01 [95% CI, 0.73-1.39]; *P* = .89, *I*^2^ = 0%). A funnel plot of the eligible studies did not show publication bias (*P* = .65) (eFigure 2 in the Supplement). Also, we found no differences between the groups when stratifying the meta-analysis by intervention method (bolus or daily supplementation) (Figure 3). Further, most of the studies observing SAEs described those as not associated with the vitamin D intervention except for Evans et al,^32^ who did not comment on a possible association.

### Hypercalcemia

Hypercalcemic values were observed in 7 studies^12,13,17,20,28,30,35^ based on the authors’ own definitions. Of these, only 1 study^28^ had a comparable control group and was available for the analysis of high-dose vitamin D (1200 IU/d) vs control (400 IU/d) on the risk of hypercalcemia. Rosendahl et al^28^ found that 32 of 364 children in the vitamin D group (8.8%) vs 27 of 362 in the control group (7.5%) developed mild hypercalcemia, defined as plasma ionized calcium levels of greater than 5.4 mg/dL (to convert to millimoles per liter, multiply by 0.25). This difference was not statistically significant according to our analysis (RR, 1.18 [95% CI, 0.72-1.93]; *P* = .51). In support, there was no statistically significant difference in mean ionized calcium concentrations between the 2 groups according to the authors, and no severe cases of hypercalcemia were registered.^28^

Mittal et al^13,17^ observed hypercalcemia cases (7 of 186) in single-bolus groups receiving 90 000, 300 000, and 600 000 IU, but no clinical adverse events were observed in children. Harnot et al^12^ reported similar results among bolus groups receiving 300 000 and 600 000 IU, with all children being asymptomatic despite hypercalcemia cases. Huynh et al^20^ reported hypercalcemia cases in both a low-dose group of 400 IU/d (7 of 36) vs a 50 000-IU bolus group (2 of 34) in which only 1 child receiving a bolus had 25(OH)D levels of greater than 100 ng/mL. Gallo et al^30^ reported that 15 of 16 children (93.7%) in a 1600-IU/d group developed 25(OH)D levels of greater than 100 ng/mL; however, no difference in total plasma calcium levels was found in this group compared with a low-dose group receiving 400 IU/d.

### Urine Ca:Cr Ratio

The urine Ca:Cr ratio was reported as abnormal in 8 studies.^10,12,14,17,22,30,31,32^ Of these, 3 studies^10,22,30^ had a comparable control group. In the study by Gallo et al,^30^ suspected hypercalciuria was reported in 1 child in each of the high-dose groups (1200 and 1600 IU/d) vs 1 case in a low-dose group (800 IU/d), but with no cases in the lowest-dose group (400 IU/d). It was not clear how the term *suspected* was defined in the study, but clinical follow-up measurements were within reference range in all children. In one of the remaining studies,^10^ borderline abnormal urine Ca:Cr ratio was observed in 9 of 104 children in the vitamin D bolus group vs 12 of 117 in the placebo group, including baseline measurements. In a study by Jensen et al,^22^ 1 of 11 children in the vitamin D bolus group vs 1 of 11 in the placebo group had a borderline abnormal urine Ca:Cr ratio.

Harnot et al^12^ reported abnormal urine Ca:Cr ratio in 300 000- and 600 000-IU bolus groups; these results are similar to those reported by Mittal et al^17^ in which cases of abnormal urine Ca:Cr ratios were reported in 90 000- and 300 000-IU bolus groups. In the study by Evans et al,^32^ increased mean urine Ca:Cr values were observed in the control group compared with the intervention group (400 vs 2000 IU/d; *P* < .001). The abnormal value reported by Mondal et al^14^ was observed before the intervention (ie, it was not associated with the intervention). Holmlund-Suila et al^31^ observed 39% of children with hypercalciuria, but with no differences between the intervention groups and no correlation with 25(OH)D levels.

### ALP, Phosphate, and PTH Findings

In studies reporting levels of ALP, these levels were within the reference range and were similar between the intervention and control groups where applicable,^15,30,32,33,35^ except for 1 study^39^ in which children in the group receiving daily doses had elevated ALP levels (n = 2), hypophosphatemia (n = 1), and hyperparathyroidism (n = 3), and children in the bolus group had elevated ALP levels (n = 2) and hyperparathyroidism (n = 1) with no statistical differences between the groups. Phosphate levels within the reference range were reported by the remaining studies.^15,32,35^ In the study by Rosendahl et al,^28^ mean PTH levels were significantly lower in the 1200 vs 400 IU/d groups both at 1 and 2 years of age.

### Risk of Bias

We used the Cochrane risk of bias tool (2019 version)^7^ to evaluate the qualities of the RCTs. Our overall risk of bias assessment showed low risk in 14 studies,^9,10,11,12,18,23,24,28,30,31,33,37,38,39^ some concerns in 4 studies,^19,20,22,29^ and high risk in 14 studies.^8,13,14,15,16,17,21,25,26,27,32,34,35,36^ This assessment is provided in detail in eTable 2 in the Supplement.

## Discussion

Based on findings reported in the 32 included RCTs in this systematic review and meta-analysis, clinical adverse events were relatively rare, and most of the reported abnormal biochemical values were not described as raising serious concern or having a clear correlation with high-dose vitamin D supplementation and excess 25(OH)D levels. Most importantly, in the 8 studies available for meta-analysis,^8,10,18,23,24,29,32,34^ there was no increased risk of SAEs among patients assigned to a high-dose vitamin D intervention, either as a bolus or daily supplement, compared with a low-dose or a placebo group. Further, we found no statistical differences in the studies reporting hypercalcemia and abnormal urine Ca:Cr ratios. Thus, our findings suggest that vitamin D supplementation in the high-dose range of 1200 to 10 000 IU/d and bolus doses to 600 000 IU to infants and preschool children to 6 years of age may be safe in both healthy children and in children with various diseases.

The studies included described high-dose vitamin D as being safe; however, many stated that their results were not sufficiently strong for making solid conclusions on safety owing to, for example, small sample sizes. The main strength of our study is the inclusion of several studies and a meta-analysis of an important safety outcome (SAEs), which allows for an overall safety assessment together with a descriptive review of reported safety end points when a meta-analysis could not be performed. Although abnormal biochemical values were still observed in some studies, and high-dose vitamin D supplementation may result in some adverse events, those reported in the studies were not considered SAEs or directly associated with the vitamin D intervention, because no excess in 25(OH)D levels was observed, which was also supported by our meta-analysis of SAEs showing no increased risk in the high-dose groups and no heterogeneity across the studies. Information about long-term clinical adverse events is generally lacking, and further RCTs with larger and more comparable study groups are needed to form a clearer image of the safety profile for high-dose vitamin D supplementation in different dose ranges.

A meta-analysis including RCTs conducted among adults given high-dose vitamin D for a minimum of 1 year showed that high-dose vitamin D supplementation did not increase the risk of total adverse events significantly, although the results showed borderline increased risk for hypercalcemia.^40^ This finding is consistent with our results and supports the notion that high-dose vitamin D intervention may be a safe treatment strategy without serious adverse events within the described range doses.

The safety outcomes assessed in this study are clinically relevant and should be investigated further because high-dose vitamin D administered to children seems to have beneficial effects on different health outcomes, such as preventing pneumonia^8,24^ and influenza.^34^ Exogenous vitamin D intoxication is a potential risk when administering high-dose supplements, and severe hypercalcemia is of greatest concern with symptoms such as vomiting, constipation, abdominal pain, dehydration, polyuria, concentration problems, and nephrocalcinosis.^41^ If the safety of high-dose vitamin D supplementation by bolus and/or daily administration is confirmed in future large-scale studies, it may be a new potential treatment strategy against several health outcomes and should result in a reevaluation of the definition of the upper levels of tolerable intake for vitamin D supplementation.

### Limitations

The main limitations of this systematic review and meta-analysis are the lack of similar intervention doses, safety and end point definitions, similarity of the included children, focused safety outcome of the studies, and a broad variation in the intervention and follow-up periods. The daily vitamin D supplementation periods ranged from 5 days until approximately 1 year in the included RCTs, which is a limitation and reduces comparability between the studies. Further, the heterogeneity in the follow-up periods is a limitation when comparing the total number of adverse events given that short-term studies may not capture the same adverse events as long-term studies. The various diagnoses of the children may have played a role when assessing adverse effects or may have resulted in different metabolism patterns. For instance, among infants with hypothyroidism, as seen in the study by Tau et al,^35^ hypercalcemia often develops because of decreased metabolism of vitamin D or increased absorption.^42^ However, we investigated clinical adverse events and abnormal biochemical measures independent of the studied population and clinical outcomes of the trials to elucidate the overall safety profile of a high-dose vitamin D intervention, which allowed for a generalization of our results. In 7 of the 8 studies included in the meta-analysis investigating the risk of SAEs, children were diagnosed with asthma, pneumonia, or very low birth weight, which could influence the generalizability of the results given that these children constitute high-risk pediatric populations. However, because these children may be considered at an increased risk for developing SAEs due to high-dose vitamin D compared with healthy children, this should not change the overall safety assessment. Furthermore, factors such as skin pigmentation, latitude, clothing, and use of sunscreen all influence the endogenous synthesis of vitamin D_3_ in the skin.^43^ In our review, the predominant racial and ethnic population represented was Asian (14 studies^8,9,11,12,13,14,17,18,19,23,24,25,29,34^); therefore, RCTs conducted among other races and ethnicities such as White individuals are warranted.

In some studies, the high-dose vitamin D intervention was given simultaneously with other treatment, which potentially could mask whether the adverse events were due to vitamin D or not. For instance, in the study by Shakiba et al,^25^ in which vitamin D was given at the same time as a routine polio vaccination, it is difficult to assess whether the potential adverse events of diarrhea and agitation seen were due to vaccination or to high-dose vitamin D. Last, many studies lacked a comparable low-dose or placebo group to allow for a meta-analysis of safety outcomes, further limiting our findings.

## Conclusions

This meta-analysis and systematic review found that adverse events occurring after vitamin D supplementation as assessed by clinical symptoms and biochemical changes were rare in the 32 RCTs included, for which high-dose vitamin D supplementation was characterized as being well tolerated overall. These results suggest that high-dose vitamin D supplementation to children aged 0 to 6 years may be a safe supplementation strategy against various health outcomes.

## Notes

Supplement. **eFigure 1.** Division of Search Terms **eFigure 2.** Funnel Plot of Studies Included in the Meta-analysis of Serious Adverse Events by High-Dose Vitamin D Interventions **eTable 1.** Search Protocol **eTable 2.** Risk of Bias Score
eReferences
