# Supplementary material for: Safety of High-Dose Vitamin D Supplementation Among Children Aged 0 to 6 Years: A Systematic Review and Meta-analysis
Source: JAMA Netw Open. 2022 Apr 14;5(4):e227410. doi: 10.1001/jamanetworkopen.2022.7410 (PMC9011124; doi:10.1001/jamanetworkopen.2022.7410)
Supplement: Supplement. — eFigure 1. Division of Search Terms eFigure 2. Funnel Plot of Studies Included in the Meta-analysis of Serious Adverse Events by High-Dose Vitamin D Interventions eTable 1. Search Protocol eTable 2. Risk of Bias Score eReferences [file jamanetwopen-e227410-s001.pdf]

Supplementary Online Content

Brustad N, Yousef S, Stokholm J, Bønnelykke K, Bisgaard H, Chawes BL. Safety of high-dose vitamin D supplementation among children aged 0 to 6 years: a systematic review and meta-analysis. *JAMA Netw Open*. 2022;5(4):e227410. doi:10.1001/jamanetworkopen.2022.7410

eFigure 1. Division of Search Terms
eFigure 2. Funnel Plot of Studies Included in the Meta-analysis of Serious Adverse Events by High-Dose Vitamin D Interventions
eTable 1. Search Protocol
eTable 2. Risk of Bias Score
eReferences

This supplementary material has been provided by the authors to give readers additional information about their work.

© 2022 Brustad N et al. *JAMA Network Open*.

eFigure 1. Division of Search Terms

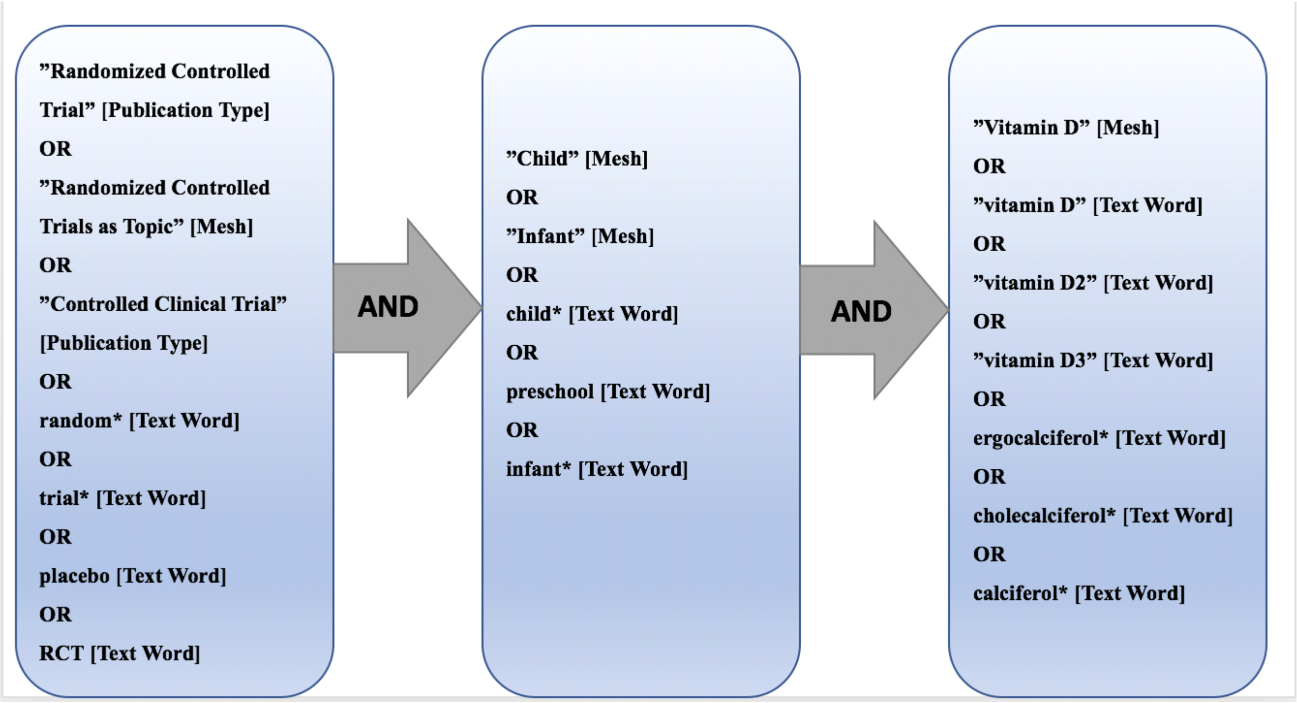

© 2022 Brustad N et al. *JAMA Network Open*.

eFigure 2. Funnel Plot of Studies Included in the Meta-analysis of Serious Adverse Events by High-Dose Vitamin D Interventions

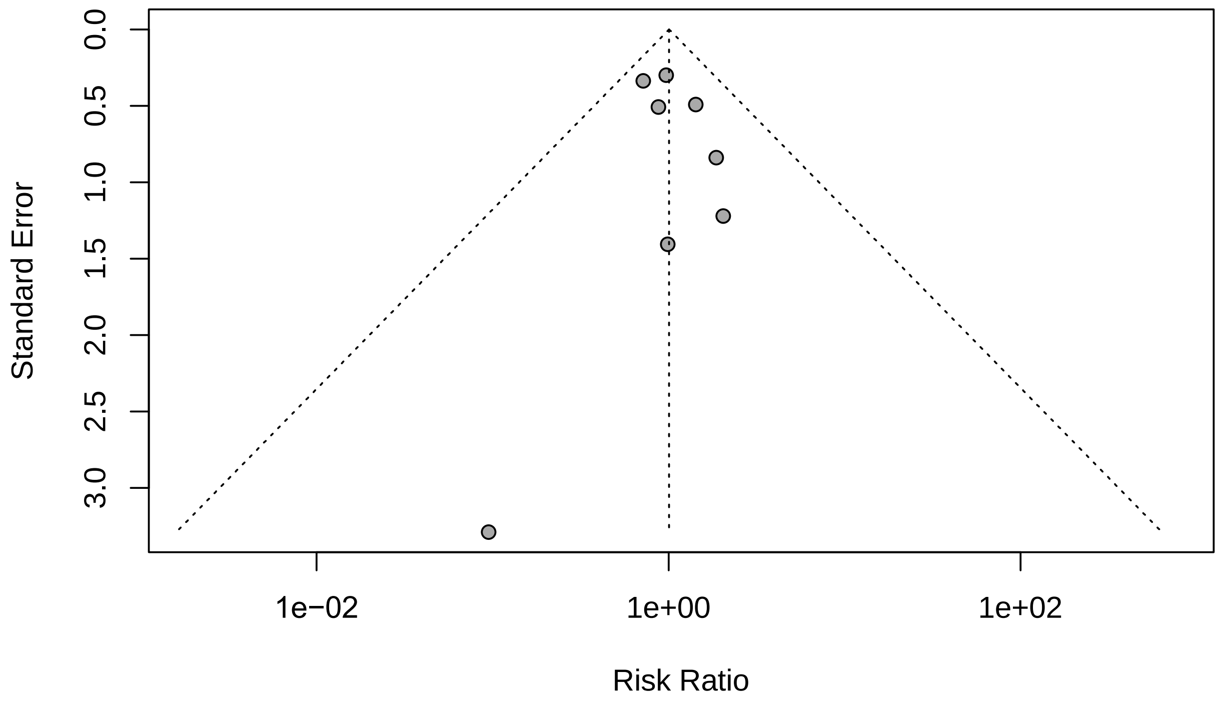

© 2022 Brustad N et al. *JAMA Network Open*.

eTable 1. Search Protocol

Database	Search Strategy	Hits
PubMed<br><br>Date:<br>24/08/2021	#1:<br>Search: **((("Randomized Controlled Trial" [Publication Type]) OR ("Randomized Controlled Trials as Topic"[Mesh])) OR ("Controlled Clinical Trial" [Publication Type])) OR ((((random*[Text Word]) OR (trial*[Text Word])) OR (placebo[Text Word])) OR (RCT[Text Word]))**<br><br>Limits: **English**	2,282,723
	#2:<br>Search: **(("Child"[Mesh]) OR ("Infant"[Mesh])) OR (((child*[Text Word]) OR (preschool[Text Word])) OR (infant*[Text Word]))**<br><br>Limits: **English**	2,528,298
	#3:<br>Search: **("Vitamin D"[Mesh]) OR ((((("vitamin D"[Text Word]) OR ("vitamin D2"[Text Word])) OR ("vitamin D3"[Text Word])) OR (ergocalciferol*[Text Word])) OR (cholecalciferol*[Text Word])) OR (calciferol*[Text Word]))**<br>Limits: **English**	84,027
	#4:<br>Search: **#1 AND #2 AND #3**<br>Limits: **English**	2,063

© 2022 Brustad N et al. *JAMA Network Open*.

eTable 2. Risk of Bias Score

Study	The randomization process	Deviations from the intended interventions *"effect of assignment to intervention"*	Missing outcome data	Measurement of the outcome	The reported results	Overall risk
Singh et al,[1] 2019	Some concerns	Some concerns	Low	Some concerns	Some concerns	High
Saleem et al,[2] 2018	Low	Low	Low	Low	Low	Low
Ducharme et al,[3] 2019	Low	Low	Low	Low	Low	Low
Moslemi et al,[4] 2018	Low	Low	Low	Low	Low	Low
Harnot et al,[5] 2016	Low	Low	Low	Low	Low	Low
Mittal et al,[6] 2014	Low	Low	Some concerns	Some concerns	Some concerns	High
Mondal et al,[7] 2014	Low	High	Some concerns	High	Low	High
Lubani et al,[8] 1989	Some concerns	High	High	High	Some concerns	High
Zeghoud et al,[9] 1994	Some concerns	High	Low	High	Some concerns	High
Mittal et al,[10] 2018	Low	Some concerns	Some concerns	Low	Some concerns	High
Gupta et al,[11] 2016	Low	Low	Low	Low	Low	Low
Somnath et al,[12] 2017	Low	Some concerns	Low	Low	Some concerns	Some concerns
Huynh et al,[13] 2016	Low	Some concerns	Low	Low	Low	Some concerns

© 2022 Brustad N et al. *JAMA Network Open*.

Moodley et al,[14] 2015	Low	High	High	Low	Some concerns	High
Jensen et al,[15] 2016	Low	Some concerns	Low	Low	Low	Some concerns
Manaseki-Holland et al,[216] 2012	Low	Low	Low	Low	Low	Low
Manaseki-Holland et al,[17] 2010	Low	Low	Low	Low	Low	Low
Shakiba et al,[18] 2010	Low	High	High	Low	Some concerns	High
Mawer et al,[19] 1986	Some concerns	High	High	Low	Some concerns	High
Moya et al,[20] 1977	High	Some concerns	Low	Low	Some concerns	High
Rosendahl et al,[21] 2018	Low	Low	Low	Low	Low	Low
Choudhary et al,[22] 2011	Low	Low	Low	Low	Some concerns	Some concerns
Gallo et al,[23] 2013	Low	Low	Low	Low	Low	Low
Holmlund-Suila et al, [24] 2012	Low	Low	Low	Low	Low	Low
Evans et al,[25] 1989	Some concerns	High	Low	Low	Some concerns	High
Aglipay et al,[26] 2017	Low	Low	Low	Low	Low	Low
Zhou et al, [27] 2018	Some concerns	High	Some concerns	Some concerns	Some concerns	High
Tau et al, [28] 1986	Some concerns	High	High	Some concerns	Some concerns	High

© 2022 Brustad N et al. *JAMA Network Open*.

Pacheco-Acosta et al,[29] 2020	Some concerns	High	Low	Low	Some concerns	High
Aldaghi et al,[30] 2020	Low	Low	Low	Low	Low	Low
Chowdhury et al,[31] 2021	Low	Low	Low	Low	Low	Low
Saluja et al,[32] 2021	Low	Low	Low	Low	Low	Low

© 2022 Brustad N et al. *JAMA Network Open*.

eReferences

1. Singh N, Kamble D, Mahantshetti NS. Effect of vitamin D supplementation in the prevention of recurrent pneumonia in under-five children. Indian J Pediatr. 2019;86(12):1105-1111. Medline:31346969 doi:10.1007/s12098-019-03025-z
2. Saleem J, Zakar R, Zakar MZ, et al. High-dose vitamin D3 in the treatment of severe acute malnutrition: a multicenter double-blind randomized controlled trial. Am J Clin Nutr. 2018;107(5):725-733. Medline:29722846 doi:10.1093/ajcn/nqy027
3. Ducharme FM, Jensen M, Mailhot G, et al. Impact of two oral doses of 100 000 IU of vitamin D3 in preschoolers with viral-induced asthma: a pilot randomised controlled trial. Trials. 2019;20(1):138. Medline:30777118 doi:10.1186/s13063-019-3184-z
4. Moslemi L, Esmaeili Dooki M, Moghadamnia AA, et al. Stoss therapy using fortified biscuit for vitamin D-deficient children: a novel treatment. Pediatr Res. 2018;84(5):662-667. Medline:30120406 doi:10.1038/s41390-018-0135-4
5. Harnot J, Verma S, Singhi S, Sankhyan N, Sachdeva N, Bharti B. Comparison of 300 000 and 600 000 IU oral vitamin-D bolus for vitamin-D deficiency in young children. Indian J Pediatr. 2017;84(2):111-116. Medline:27683282 doi:10.1007/s12098-016-2233-9
6. Mittal H, Rai S, Shah D, et al. 300 000 IU or 600 000 IU of oral vitamin D3 for treatment of nutritional rickets: a randomized controlled trial. Indian Pediatr. 2014;51(4):265-272. Medline:24825262 doi:10.1007/s13312-014-0399-76
7. Mondal K, Seth A, Marwaha RK, et al. A randomized controlled trial on safety and efficacy of single intramuscular versus staggered oral dose of 600 000 IU vitamin D in treatment of nutritional rickets. J Trop Pediatr. 2014;60(3):203-210. Medline:24401754 doi:10.1093/tropej/fmt1057
8. Lubani MM, al-Shab TS, al-Saleh QA, et al. Vitamin-D–deficiency rickets in Kuwait: the prevalence of a preventable disease. Ann Trop Paediatr. 1989;9(3):134-139. Medline:2475056 doi:10.1080/02724936.1989.11748616
9. Zeghoud F, Ben-Mekhbi H, Djeghri N, Garabédian M. Vitamin D prophylaxis during infancy: comparison of the long-term effects of three intermittent doses (15, 5, or 2.5 mg) on 25-hydroxyvitamin D concentrations. Am J Clin Nutr. 1994;60(3):393-396. Medline:8074071 doi:10.1093/ajcn/60.3.393
10. Mittal M, Yadav V, Khadgawat R, Kumar M, Sherwani P. Efficacy and safety of 90 000 IU versus 300 000 IU single dose oral vitamin D in nutritional rickets: a randomized controlled trial. Indian J Endocrinol Metab. 2018;22(6):760-765. Medline:30766814 doi:10.4103/ijem.IJEM_84_18
11. Gupta P, Dewan P, Shah D, et al. Vitamin D supplementation for treatment and prevention of pneumonia in under-five children: A Randomized Double-blind Placebo Controlled Trial. Indian Pediatr. 2016;53(11):967-976. Medline:27889723 doi:10.1007/s13312-016-0970-5
12. Somnath SH, Biswal N, Chandrasekaran V, Jagadisan B, Bobby Z. Therapeutic effect of vitamin D in acute lower respiratory infection: a randomized controlled trial. Clin Nutr ESPEN. 2017;20:24-28. Medline:29072165 doi:10.1016/j.clnesp.2017.02.003
13. Huynh J, Lu T, Liew D, et al. Vitamin D in newborns: a randomised controlled trial comparing daily and single oral bolus vitamin D in infants. J Paediatr Child Health. 2017;53(2):163-169. Medline:27670154 doi:10.1111/jpc.13338
14. Moodley A, Spector SA. Single high-dose vitamin D at birth corrects vitamin D deficiency in infants in Mexico. Int J Food Sci Nutr. 2015;66(3):336-341. Medline:25582182 doi:10.3109/09637486.2014.992006
15. Jensen ME, Mailhot G, Alos N, et al. Vitamin D intervention in preschoolers with viral-induced asthma (DIVA): a pilot randomised controlled trial. Trials. 2016;17(1):353. Medline:27456232 doi:10.1186/s13063-016-1483-1
16. Manaseki-Holland S, Maroof Z, Bruce J, et al. Effect on the incidence of pneumonia of vitamin D supplementation by quarterly bolus dose to infants in Kabul: a randomised controlled superiority trial. Lancet. 2012;379(9824):1419-1427. Medline:22494826 doi:10.1016/S0140-6736(11)61650-4
17. Manaseki-Holland S, Qader G, Isaq Masher M, et al. Effects of vitamin D supplementation to children diagnosed with pneumonia in Kabul: a randomised controlled trial. Trop Med Int Health. 2010;15(10):1148-1155. Medline:20723187 doi:10.1111/j.1365-3156.2010.02578.x
18. Shakiba M, Sadr S, Nefei Z, Mozaffari-Khosravi H, Lotfi MH, Bemanian MH. Combination of bolus dose vitamin D with routine vaccination in infants: a randomised trial. Singapore Med J. 2010;51(5):440-445. Medline:20593151
19. Mawer EB, Stanbury W, Robinson MJ, James J, Close C. Vitamin D nutrition and vitamin D metabolism in the premature human neonate. Clin Endocrinol (Oxf). 1986;25(6):641-649. Medline:3652468 doi:10.1111/j.1365-2265.1986.tb03619.x
20. Moya M, Beltran J, Colomer J. Therapeutic and collateral effects of 25-hydroxycholecalciferol in vitamin D deficiency. Eur J Pediatr. 1977;127(1):49-55. Medline:23950 doi:10.1007/BF00465565
21. Rosendahl J, Valkama S, Holmlund-Suila E, et al. Effect of higher vs standard dosage of vitamin D3 supplementation on bone strength and infection in healthy infants: a randomized clinical trial. JAMA Pediatr. 2018;172(7):646-654. Medline:29813149 doi:10.1001/jamapediatrics.2018.0602
22. Choudhary N, Gupta P. Vitamin D supplementation for severe pneumonia—a randomized controlled trial. Indian Pediatr. 2012;49(6):449-454. Medline:21992858 doi:10.1007/s13312-012-0073-x

© 2022 Brustad N et al. *JAMA Network Open*.

23. Gallo S, Comeau K, Vanstone C, et al. Effect of different dosages of oral vitamin D supplementation on vitamin D status in healthy, breastfed infants: a randomized trial. JAMA. 2013;309(17):1785-1792. Medline:23632722 doi:10.1001/jama.2013.3404
24. Holmlund-Suila E, Viljakainen H, Hytinantti T, Lamberg-Allardt C, Andersson S, Mäkitie O. High-dose vitamin d intervention in infants—effects on vitamin D status, calcium homeostasis, and bone strength. J Clin Endocrinol Metab. 2012;97(11):4139-4147. Medline:22933541 doi:10.1210/jc.2012-1575
25. Evans JR, Allen AC, Stinson DA, et al. Effect of high-dose vitamin D supplementation on radiographically detectable bone disease of very low birth weight infants. J Pediatr. 1989;115(5, pt 1):779-786. Medline:2809913 doi:10.1016/S0022-3476(89)80662-6
26. Aglipay M, Birken CS, Parkin PC, et al; TARGet Kids! Collaboration. Effect of high-dose vs standard-dose wintertime vitamin D supplementation on viral upper respiratory tract infections in young healthy children. JAMA. 2017;318(3):245-254. Medline:28719693 doi:10.1001/jama.2017.8708
27. Zhou J, Du J, Huang L, Wang Y, Shi Y, Lin H. Preventive effects of vitamin D on seasonal influenza A in infants: a multicenter, randomized, open, controlled clinical trial. Pediatr Infect Dis J. 2018;37(8):749-754. Medline:29315160 doi:10.1097/INF.0000000000001890
28. Tau C, Garabedian M, Farriaux JP, Czernichow P, Pomarede R, Balsan S. Hypercalcemia in infants with congenital hypothyroidism and its relation to vitamin D and thyroid hormones. J Pediatr. 1986;109(5):808-814. Medline:3490559 doi:10.1016/S0022-3476(86)80698-9
29. Pacheco-Acosta J, Pizarro F. Effect of vitamin D supplementation as a single dose on the nutritional status of vitamin D [in Spanish and English]. Rev Chil Pediatr. 2020;91(5):684-690. Medline:33399632 doi:10.32641/rchped.v91i5.1402
30. Aldaghi M, Tehrani H, Karrabi M, Abadi FS, Sahebkar M. The effect of multistrain synbiotic and vitamin D3 supplements on the severity of atopic dermatitis among infants under 1 year of age: a double-blind, randomized clinical trial study. J Dermatolog Treat. Published online June 26, 2020. Medline:32530339 doi:10.1080/09546634.2020.1782319
31. Chowdhury F, Shahid ASMSB, Tabassum M, et al. Vitamin D supplementation among Bangladeshi children under-five years of age hospitalised for severe pneumonia: a randomised placebo controlled trial. PLoS One. 2021;16(2):e0246460. Medline:33606713 doi:10.1371/journal.pone.0246460
32. Saluja RK, Dewan P, Gomber S, Madhu SV, Bhat S, Gupta P. Low dose depot oral vitamin D3 v daily oral vitamin D3 for treating nutritional rickets: a randomised clinical trial. Br J Nutr. Published online July 19, 2021. doi:10.1017/S0007114521002713

© 2022 Brustad N et al. *JAMA Network Open*.
